# A comparative evaluation of two subharmonic-aided pressure estimation (SHAPE) analysis methods

**DOI:** 10.1016/j.ultras.2025.107840

**Published:** 2025-10-01

**Authors:** Hailee Mayer, Priscilla Machado, Trang Vu, Annalisa Berzigotti, Jaume Bosch, Elton Dajti, Antonina Antonenko, Kirk Wallace, Flemming Forsberg

**Affiliations:** a Thomas Jefferson University, Department of Radiology, Philadelphia, PA, USA; b Drexel University, School of Biomedical Engineering, Science, and Health Systems, Philadelphia, PA, USA; c University of Bern, Department of Visceral Surgery and Medicine, Inselpital, Bern University Hospital, Bern, Switzerland; d Department of Medical and Surgical Sciences, University of Bologna, Italy; e GE HealthCare, Niskayuana, NY, USA

**Keywords:** Ultrasound, Portal hypertension, Subharmonic, UCAs, SHAPE

## Abstract

Pressure measurement in clinical practice is a valuable tool for diagnostic evaluation, especially in diseases such as portal hypertension. Currently, portal pressures are measured by invasive hepatic vein catheterization, posing risks to patients, and limiting how often measurements can be obtained. Subharmonic-aided pressure estimation (SHAPE) is an ultrasound-based technique that leverages ultrasound contrast agents (UCAs) to estimate changes in hydrostatic pressure. UCAs act as nonlinear oscillators when exposed to high enough acoustic pressures (typically > 200 kPa) and produce significant energy components ranging from subharmonics to higher harmonics. Tissues do not generate significant subharmonic components, thus making it the optimal component for SHAPE. SHAPE is performed using subharmonic imaging complimented by an acoustic pressure optimization algorithm to induce the highest sensitivity to hydrostatic pressure in the UCAs. Traditionally, multiple acquisitions of contrast clips are used to identify the subharmonic amplitude and calculate SHAPE parameters, but recently, a faster method of SHAPE has been proposed, utilizing only the acoustic pressure optimization algorithm to obtain subharmonic amplitudes. The objective of this work is to compare the two methods, SHAPE and fast-SHAPE, in the same patients, to explore the utility of fast-SHAPE.

## 1. Introduction

Subharmonic-aided pressure estimation (SHAPE) is an ultrasound-based technique that measures changes in hydrostatic pressure by leveraging the oscillations of ultrasound contrast agents (UCAs). UCAs consist of microbubbles (MBs) ranging from 1 to 10 μm in size and act as vascular tracers following IV injection or infusion, and will oscillate when exposed to an acoustic field [1]. More specifically, when UCAs are exposed to high enough acoustic pressures, typically above 200 kPa, their oscillations contain nonlinear frequency components, ranging from subharmonics to ultraharmonics. The subharmonic component is of particular interest for SHAPE as there is minimal subharmonic generation from tissues, and therefore, when performing SHAPE, the acquired signal is specific to the UCAs, and tissues can be ignored [1–3]. Moreover, bubbly media has a nonlinearity index (B/A) that is 1–2 orders of magnitude greater than tissues and received echoes can cause overlap in higher harmonics [4,5]. SHAPE has been studied by our group and others both *in vitro* and *in vivo* and has shown excellent correlations between subharmonic amplitude and changes in hydrostatic pressure [6–23]. The generation of subharmonic signal by UCAs has three distinct phases; in the occurrence phase the subharmonic amplitude cannot be distinguished from noise, in the growth phase, the subharmonic amplitude grows rapidly with increasing acoustic pressure until the signal plateaus and the saturation phase is reached [2,3]. The majority of UCAs have an inverse linear subharmonic response to hydrostatic pressure [6], and the sensitivity of this trend (i.e., the slope of the linear regression model) can be optimized by calibrating the acoustic power on an individual basis so it corresponds to the inflection point of the growth phase [7,17]. This acoustic power will produce the highest sensitivity for SHAPE.

Currently, SHAPE is commercially available on the Resona family of scanners by Mindray and the LOGIQ E10 system by GE HealthCare. The latter system was used by Kuroda et al. in their 2024 study evaluating SHAPE’s ability to identify patients with high-risk esophagogastric varices (EVs) [22]. More specifically, this study recruited patients with chronic liver disease that were undergoing endoscopic screening for EVs, which are a serious complication of clinically significant portal hypertension and is marked by enlarged veins within the esophagus. As varices grow and worsen, the likelihood of a bleeding event increases, thus early detection of EVs, especially those at high-risk for bleeding is crucial for treatment. Currently, endoscopic screening is the reference standard, however, 60 % of patients that undergo endoscopic screening do not have varices. Kuroda showed that SHAPE can be used as a reliable screening tool for EVs in these patients, compared to endoscopic findings. In their study, SHAPE was performed using the UCA Sonazoid (GE HealthCare) at a dose of 0.0075 mL/kg. A C1-6-D probe and the LOGIQ E10 were used for SHAPE acquisitions with an acoustic power optimization algorithm consisting of a 20 s sweep during which the mechanical index (MI) was increased from 0 to 0.50 in intervals of 0.05 every 0.5 s (while patients were requested to hold their breath). One frame is collected at each MI at a given frame rate (in these studies around 10 Hz) and a time-intensity-curve (TIC) is used to plot the subharmonic signal at each MI. The resulting sigmoidal curve is used to identify the optimal acoustic pressure, as the point of the maximum slope identified using the derivative of the optimization TIC.

In all SHAPE studies to date, the acoustic power optimization algorithm is only used initially to identify the optimal MI, and then 3–5 5 s cine clips are acquired to obtain the SHAPE values [8–11]. However, Kuroda introduced a new approach by acquiring the subharmonic amplitude of the hepatic and portal vein directly from the time-intensity-curve (TIC) generated from the acoustic pressure calibration [22]. A region of interest (ROI) with a 10 mm diameter was placed in the portal and hepatic veins at the same depth. The inflection point of the curve generated for the portal vein was used to identify the optimum power, and the signals in the hepatic minus the portal vein (HV and PV, respectively) at the optimum power was calculated as the SHAPE gradient measurement. This technique was performed in 111 patients with liver cirrhosis to determine SHAPE’s ability to discriminate between null-risk, low-risk, and high-risk of esophageal varices resulting in an area under the curve (AUC) of 0.92 (95 % CI: 0.87–0.97) for SHAPE’s ability to discriminate the high-risk group from the others. The author’s concluded that there is compelling evidence that this method of SHAPE could be used as a precise, noninvasive method for discriminating high-risk esophageal varices in patients with liver cirrhosis, and that broader patient populations should be studied to confirm its effectiveness [22].

As previously mentioned, SHAPE is traditionally performed by first calibrating the acoustic power and then acquiring 3–5 5 s cine clips at the optimal acoustic pressure. Off-line, the cine-clips are analyzed using TIC analysis to calculate the SHAPE gradient (although this processing could obviously be moved onto the scanner itself). Hence, the aim of this study was to compare Kuroda’s method of SHAPE acquisition, which will be referred to as “fast-SHAPE” and the traditional cine-clip method of SHAPE (referred to as “SHAPE”) in the same patients.

## 2. Methods

**Fig. 1.**
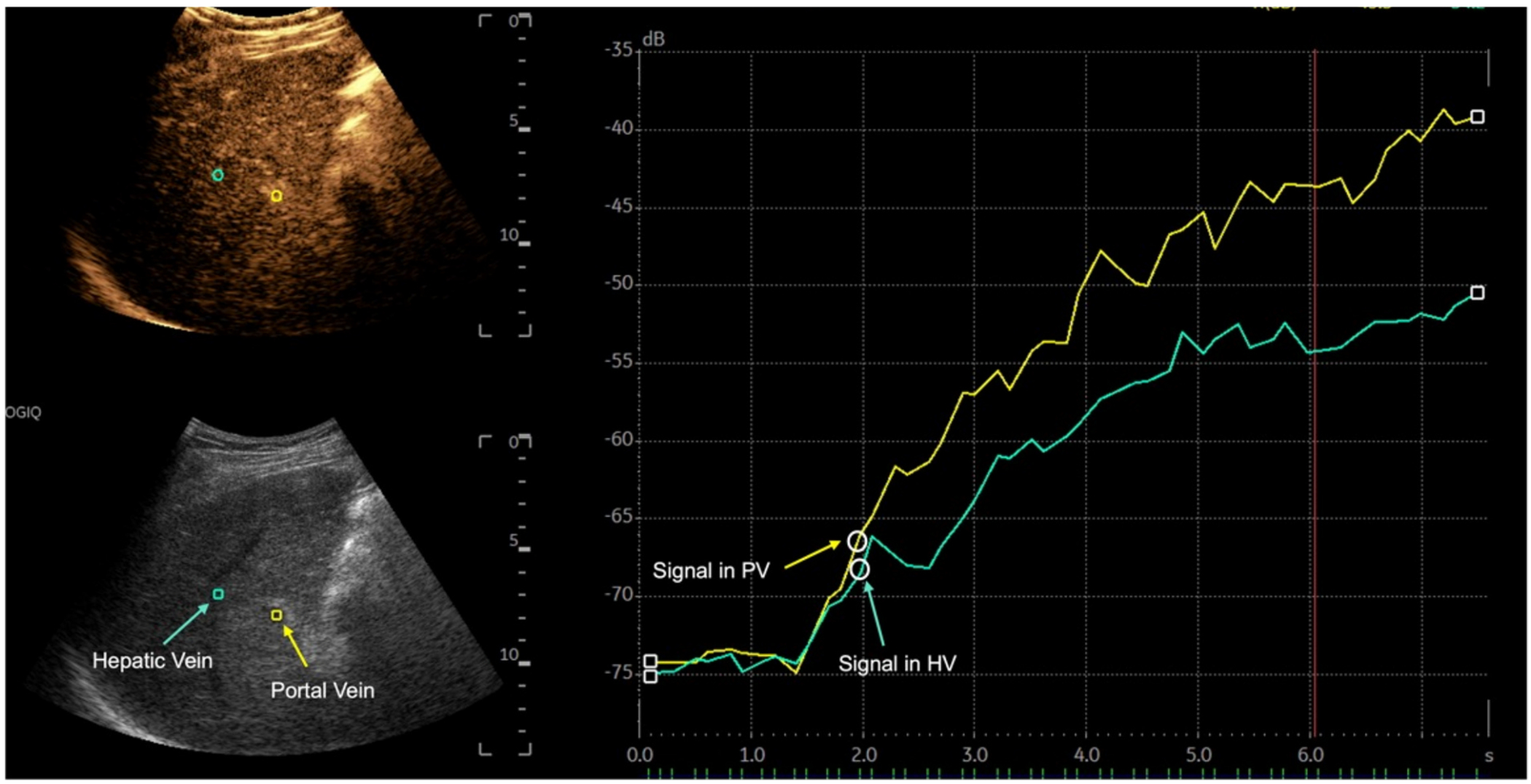
Acoustic Power Optimization Time-Intensity-Curve (TIC) Used to Obtain Subharmonic Amplitudes. Dual-imaging showing B-mode (bottom) and contrast (top) is shown on the left side of the figure. On the right is the accompanying TIC used to obtain the subharmonic amplitudes within the Portal Vein (PV) and Hepatic Vein (HV) to calculate the SHAPE gradient.

A sub-set of 28 patients with clinically significant portal hypertension that have been enrolled in an on-going IRB- and FDA-approved study (NCT05470205; IND 124,465) of SHAPE in patients have had their data analyzed for this study. Patients received SHAPE exams using Sonazoid and a LOGIQ E10 system. The system was equipped with an acoustic power optimization algorithm functionally similar to what was employed be Kuroda. This version of the optimization algorithm runs on average for 4–7 s, as opposed to 20 s, although the MI is still swept from 0 to 0.50. Aside from identifying the optimal MI, the subharmonic amplitude of the PV and HV was also acquired from the sigmoidal curve generated from optimization, to calculate the fast-SHAPE gradient, as shown in Fig. 1. Following this acquisition, 3–5 5 s cine clips were acquired with the PV and HV at the same depth, and were subsequently analyzed offline, by placing an ROI in the portal and hepatic vein at the same depth and then averaging the signal in the veins over the 3–5 clips. Finally, the SHAPE gradient was calculated as the average hepatic subharmonic signal minus the average portal subharmonic signal [8,9].

### 2.1. Data/statistical analysis

First, a paired *t*-test was employed to explore the average differences between the two methods, then simple linear regression was used to further understand the correlation between fast-SHAPE and SHAPE. Finally, a Bland-Altman comparison was run to visually investigate the differences between the two methods and any bias that may exist [24–26]. Of the 28 subjects, 6 had hepatic venous pressure gradient (HVPG) measurements available (the invasive, clinical reference standard for portal hypertension), and linear regression was employed to compare fast-SHAPE’s correlation with HVPG relative to that of SHAPE.

## 3. Results

### 3.1. Fast-SHAPE and SHAPE

**Fig. 2.**
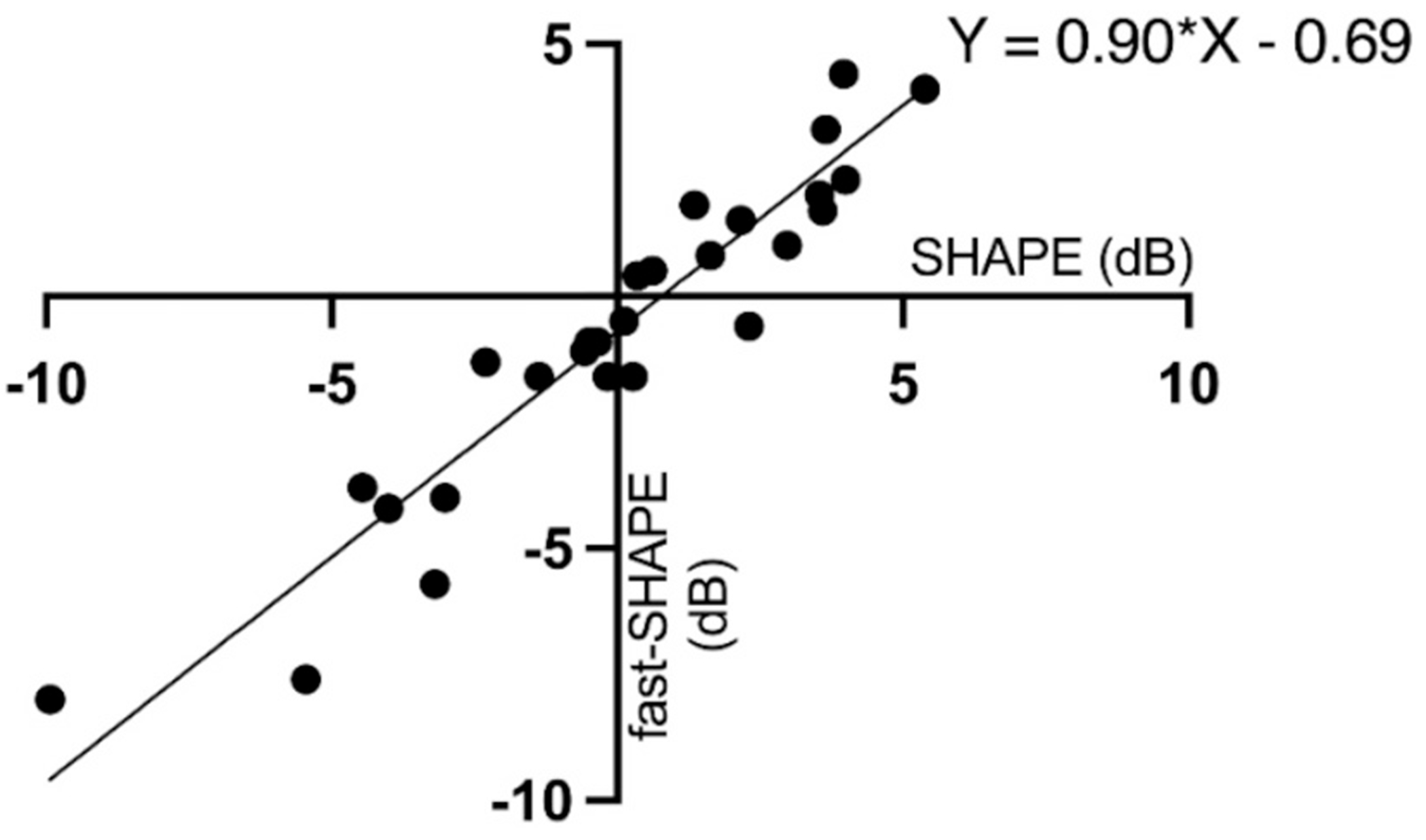
Simple Linear Regression Plot of SHAPE and fast-SHAPE. Fig. 2 shows the linear regression plot to determine the correlation between SHAPE and fastSHAPE in 28 patients, which resulted in a slope of 0.90 and an r^2^ of 0.90, indicating a moderately strong correlation.

**Fig. 3.**
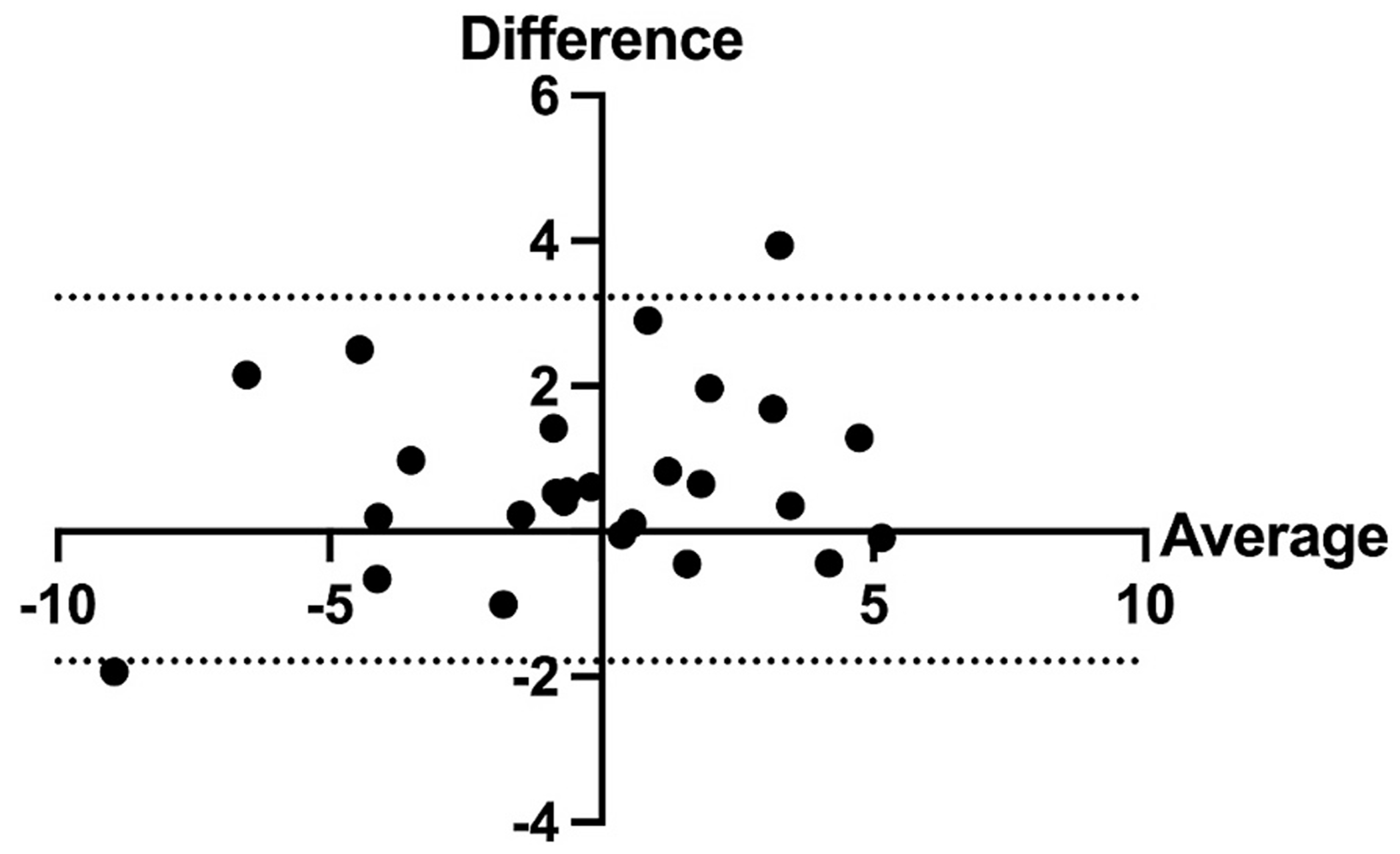
Bland-Altman Plot for Comparison of Methods. Fig. 3 shows the Bland-Altmann plot for the comparison of SHAPE and fast-SHAPE. This analysis resulted in a bias of 0.72 (standard deviation: 1.12) with 95 % limits of agreement ranging from −1.48 to 2.91 dB.

The average subharmonic amplitude in the fast-SHAPE group was 0.20 ± 3.55 dB (95 % CI: −1.17 to 1.58), while the SHAPE group’s average was −0.51 ± 3.34 dB (95 % CI: −1.81 to 0.78), with a mean difference of −0.72 dB (p = 0.02). Although the two methods were statistically significantly different in their mean values, linear regression analysis resulted in a slope of 0.90 (95 % CI: 0.77 to 1.0) and an r^2^ value of 0.90, indicating a strong correlation between the two methods, as shown in Fig. 2. Finally, the Bland-Altman analysis resulted in a bias of 0.72 (standard deviation of bias: 1.12) with 95 % limits of agreement ranging from −1.48 to 2.91, as shown in Fig. 3. Additional analysis was performed to understand the variability within the SHAPE gradient measurements (i.e., over the 3–5 5 s clips). On average, there was 1.34 dB of variability around the SHAPE gradient.

### 3.2. Fast-SHAPE and HVPG

**Fig. 4.**
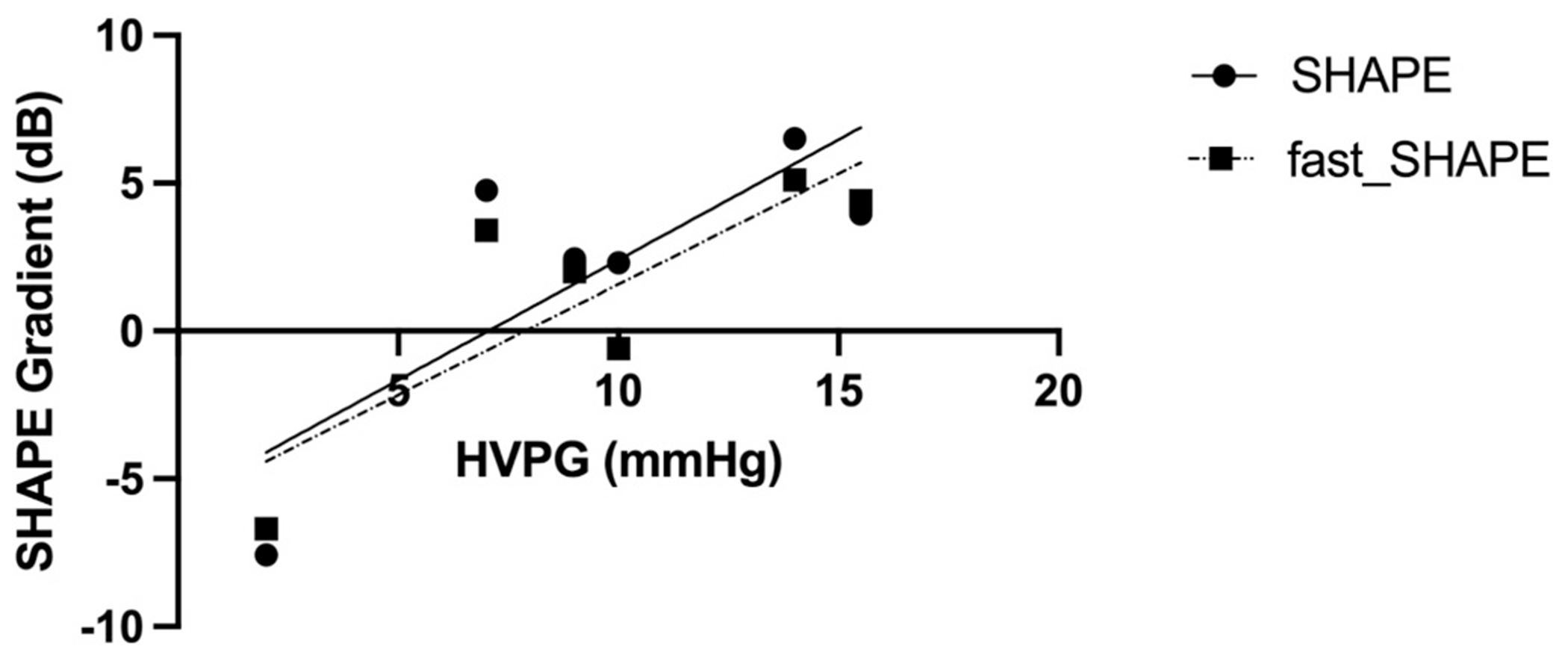
Linear Regression between HVPG and SHAPE and fast-SHAPE. Seen is the linear regression plot for 6 patients who had SHAPE, fast-SHAPE, and HVPG information available. Pearson correlation coefficients for fast-SHAPE and SHAPE were 0.83 and 0.80, respectively (p < 0.05).

In the 6 patients with HVPG information available, 3 patients had clinically significant portal hypertension (HVPG > 10 mmHg), 2 patients had non-clinically significant portal hypertension (5 < HVPG < 10 mmHg), and one patient had normal HVPG (HVPG < 5 mmHg). Linear regression resulted in a slope of 0.75 dB/mmHg for fast-SHAPE and 0.81 for SHAPE. Both methods strongly correlated with HVPG, with Pearson correlation coefficients of 0.83 and 0.80 for fast-SHAPE and SHAPE respectively (p < 0.05). These regression models can be seen in Fig. 4.

## 4. Discussion

Although a statistically significant difference was seen between fast-SHAPE and SHAPE, there was also a strong correlation between the two methods. The average difference between the two methods was −0.72 dB, but the impact of this difference is challenging to interpret. On average, there was 1.34 dB of variability around the SHAPE gradient, meaning the average difference of −0.72 dB between the two methods would be included, on average, within that range, thus the inherent variability of SHAPE measurements would mitigate the slight differences in the two methods. Therefore, the differences observed in SHAPE and fast-SHAPE in this study was not clinically relevant in this group of patients.

Gupta et al. showed SHAPE to be a reliable noninvasive evaluator of portal hypertension in 125 patients who underwent HVPG measurements. Their study achieved a sensitivity of 91 % (95 % CI: 88 % to 93 %) and a specificity of 82 % (95 % CI: 75 % to 85 %) for SHAPE to identify patients with clinically significant portal hypertension [9]. Although the results presented by Kurodar et al. and Gupta et al. are promising, but without having the cine-clips to assess the flow of UCAs to the portal venous system, cases in which SHAPE are less reliable or not feasible may not be identified. There are situations in which portal pressures can be altered from hemodynamic changes caused by the patient’s disease and treatment. For example, patients with intrahepatic portosystemic shunts have had their normal flow of blood from the digestive track to the liver through the portal vein rerouted to go directly to the hepatic vein. These shunts are often used to treat portal hypertension, but they may underestimate the severity of disease in tests such as hepatic venous portal gradient (HVPG), due to this reconstructed pathway [27]. Further, the development of collateral vessels is a characteristic of impaired portal hemodynamics, and portal blood flow is reduced because of these collaterals, and SHAPE values may not accurately reflect degree of disease in these patients [27,28]. Having access to the cine-clips from which the subharmonic is acquired allows the researcher to visually assess the performance of SHAPE, taking note of homogenous bubble flow into the veins, adequate signal within each vein, and whether or not the patient moved or breathed during acquisition, which can also impact SHAPE measurements. In traditional SHAPE, certain frames or clips that may not have adequate signal or motion present can be removed, but in fast-SHAPE, there is no way of knowing whether these problems occurred.

Aside from disease etiology impacting portal hemodynamics, and therefore impacting SHAPE measurements, there is inherent variability within SHAPE measurements, as exemplified by the average 1.34 dB of variability around the SHAPE gradient over the 3–5 cine clips. With the traditional SHAPE method, this variability can be mitigated by averaging, but in fast-SHAPE, this inherent variation is ignored, which most likely leads to the differences observed between the two methods. Kuroda showed fast-SHAPE to be a reliable test for distinguishing between null-risk, low-risk, and high-risk EVs patients with average SHAPE gradients of −7.0 dB, −4.4 dB, and −2.0 dB for the null-risk, low- risk, and high-risk group, respectively, with significant differences between all groups (p < 0.05) [22]. These results are promising, but it is not clear yet how reliable fast-SHAPE is in a broader population of liver disease patients. Strong correlation was seen between fast-SHAPE and HVPG for the subset of patients with HVPG information available (n = 6), as shown in Fig. 4, but a larger sample of patients with HVPG information available should be investigated to further support this correlation. The small difference seen between the SHAPE and fast-SHAPE may be trivial for a binary outcome such as EVs, but further studies are needed to determine the utility of fast-SHAPE for other measures, such as HVPG estimation, interstitial fluid pressure, or intracranial pressure [8,9,13,15]. Finally, fast-SHAPE would clearly not be useful in clinical situations where real-time, quantitative pressure estimation is required – such as in echocardiography [11,23].

## 5. Conclusions

In conclusion, fast-SHAPE has been showed to be a reliable tool to distinguish between patients at different risk for EVs. In situations where real-time quantitative pressures are required, fast-SHAPE’s utility is limited, but with EVs or other binary outcomes, fast-SHAPE may be quicker and simpler than SHAPE. Further studies of various etiologies should be conducted to establish a more complete understanding of fast-SHAPE’s utility compared to traditional SHAPE.
